# COVID-19: facts and failures, a tale of two worlds

**DOI:** 10.1007/s10654-020-00692-7

**Published:** 2020-11-22

**Authors:** Sergio Alejandro Gómez-Ochoa, Oscar H. Franco

**Affiliations:** 1 Public Health and Epidemiological Studies Group, Cardiovascular Foundation of Colombia, Floridablanca, Colombia; 2 Professor of Epidemiology and Public Health, Institute of Social and Preventive Medicine (ISPM), University of Bern, Mittelstrasse 43, 3012 Bern, Switzerland

The new coronavirus disease of 2019 (COVID-19) pandemic has resulted in a major public health crisis and a colossal political and communication challenge for governments, media, and citizens around the world [1]. From the beginning of the pandemic, the generalized lack of knowledge about this new disease generated an enormous debate across countries regarding the optimal preventive strategies and policies to mitigate its spread [2]. The truth is that even today, after almost a year from the first COVID-19 case report, critical aspects of the disease are still unknown; however, the negative impact of the SARS-CoV-2 pandemic has driven the attention of the world towards the governments and institutions, looking for answers to the question: “Could we have done it better?” [3]. In this context, several studies assessing the excess deaths during the last months compared to previous years have shown that despite the preventive measures taken, COVID-19 has left a deadly footprint in most regions worldwide [4]. However, this mark has not been the same for all countries, and these differences are being currently analyzed to comprehend, which interventions seemed to be successful and which failed its purpose [5, 6]. In this commentary, we will discuss the scenarios of two different countries: Denmark and the United States, by analyzing the results from two studies recently published in the European Journal of Epidemiology [7, 8], highlighting the different approaches taken to manage the pandemic by the two governments, and subsequently, the results so far.

Although how the disease takes place and spreads depends largely in the nature of the disease as well as the healthcare system, population characteristics and policies formulated, it’s been commonly reported that around 20% of all cases tend to require hospitalization, while 5–10% of the cases might require intensive care treatment [9]. To delay the spread of the disease and avoid collapse of the healthcare systems, governments have been obliged to implement lockdowns. By mid-April 2020, almost half of the world’s population was facing confinement due to the uncontrollable evolution of the pandemic [10, 11]. However, alongside the direct impact of the virus, lockdowns pose a critical challenge for populations mental health and could have severely harmed the nations’ economies, causing, for example, a fall of the United Kingdom’s GDP by 20.4% in April 2020 and an increase in the United States (U.S.) unemployment rate of around 200% [12, 13]. This cocktail of unfortunate events has pushed citizens of many countries to the limit, looking for answers to the high cost the society has paid during this pandemic, and opposing further restrictive measures even violently as recently observed in European nations facing a second wave.

In this context, the U.S. represents one of the most heavily affected nations by the SARS-CoV-2 pandemic and one of the most criticized due to the government’s management of the current situation [14, 15]. At first, despite the early implementation of a travel ban for non-U.S. travelers from China, the federal government did not implement a mandatory symptom screening or SARS-CoV-2 detection test at entry, nor required a quarantine period for individuals arriving not only from China but from other countries with confirmed viral circulation such as Italy and Spain [16]. The result of this was the introduction of several viral lineages circulating in Europe, which became the great majority of the circulating virus in the U.S [17, 18]. On the other hand, a totally different picture was observed in Denmark. In contrast with the U.S. situation, the Danish government was among the first European countries to take firm actions against the SARS-CoV-2 spread, declaring one of the earliest national lockdown and borders closure just 2 weeks after the first case was reported in the country [19]. The most immediate observed consequence of this early intervention was the reduction of the prevalence of flu in this country, with a significant drop in the percentage of positive tests when comparing the week in which the preventive measures were issued and the immediately next epidemiological week (20% vs. 7%, respectively), despite the similar number of tests performed in the 2 weeks [7, 20]. Furthermore, compared to its neighbor countries, Denmark has shown a more favorable trend regarding the number of new cases of COVID-19 from the beginning of the pandemic, allowing an earlier gradual return to labors such as ordinary hospital care [21, 22].

However, appraisals of the responses must look beyond the number of cases alone, as the important contrasts in mortality rates between regions merit a more in-depth analysis. In this context, estimates of excess deaths provide an appropriate manner to approach the excess mortality due to COVID-19 [23]. In the study of Hanage et al., an estimated range of 286,425–333,906 excess number of deaths was calculated using U.S. mortality data as of September 12, 2020. Furthermore, the authors estimated an annual excess age-standardized death rate of 91.6 per 100,000 p-y (95% CI 89.3–93.9) after comparing age-standardized mortality rates for 2020 vs. 2015–2019. On the other hand, the study of Mills et al. assessed the effect of lockdown on all-cause mortality in Denmark, finding a similar prevalence of comorbidities among deceased patients compared to previous years and highlighting the lack of an increase in the mortality rates during 2020 compared to the same period during 2015–2019 [7].

The observed differences between these two developed nations have a multi-factorial origin, including geographical aspects such as Denmark’s peninsular condition and its low population density (137 people per km^2^); however, these may not be the sole explaining factors. The government’s early lockdown and border closing and the Danish health care system’s high quality and equity may have been crucial for achieving this success in pandemic control [24, 25]. On the other hand, the lack of timely preventive measures implementation by the U.S. federal government and the deep racial/ethnic inequities in the healthcare system access and overall health status has led to the actual situation in which, the spread of COVID-19 across the country seems beyond control [26, 27].

However, any analysis in this theme would be incomplete without considering each context’s cultural and social factors. The trust in the government and other societal factors may represent one of the main conditions that had led to the Danish success [28]. The Danish citizens have proven to be less prone to believing in conspiracy theories surrounding the COVID-19 pandemic handling [29]. At the same time, they tend to trust and strongly support the government and politicians, as reflected by the government’s preventive measures’ rapid application [30]. Furthermore, the Danish culture tends to respect some social distancing by default, as they frequently respect the distance between each individual in public settings (public transportation, supermarkets, etc.) and usually have less physical contact in terms of hugs, handshakes, or kisses [29, 31]. Finally, the Danish concept of “*hygge*,” which reflects happiness through the delight of simple things of daily life and the enjoyment of sharing time in their houses with their families, may play an essential role in this COVID-19 crisis, as it may have made easier for the Danes to endure the lockdown [29, 30].

On the other hand, a simultaneous epidemic of misinformation and conspiracy ideas have flooded the U.S. since the beginning of the pandemic, as studies have revealed that almost a third of the Americans endorse conspiracy theories surrounding the COVID-19 management [31–34]. These conspiracy beliefs and their associations with perceptions of lockdowns, facemasks use, and social distancing measures harm has contributed to widespread confusion and skepticism towards many government recommendations across the country [35, 36]. Furthermore, Americans tend to spend time outside their homes with family and friends, increasing their exposure to the virus compared to the Danes [37]. All circumstances fueled by a bitter electoral process with the strengthening of differences, beliefs and behaviors caused by a bipartidism that has sowed further divisions, differences in opinions and altercations, leading to divergence rather than convergence and solidarity, factors so desperately needed in times of a pandemic.

To conclude, what is clear for everyone is that every severe case and death due to COVID-19 cannot be prevented; however, understanding the different factors influencing the transmission of the virus and applying national policies congruent to these taking into account the inherent sociocultural factors, and as promptly as possible, are crucial actions for the effective management of the SARS-CoV-2 crisis worldwide. Politicians in every country should be encouraged to follow scientific recommendations and learn from the experience of other countries that have been successful in managing the pandemic in order to promote better control of the situation. This will be the key to creating an optimal setting for the vaccines’ arrival and the progressive complete re-opening of our society. As the pandemic evolves, it’s never too late to prepare, anticipate and learn; and even if a solution comes soon; highly valuable lessons will be derived from this challenging experience, lessons desperately needed because the question is not whether we will have another pandemic, but when.

In the meantime, introspective and detailed evaluations of 2020 decisions and management of the crisis are desperately needed to identify what works and learn from what doesn’t. Parallelly, of paramount importance are immediate actions to improve the current response and health status of the population. Beyond disease and mortality, economic aspects as well as the mental health of the population ought to be critical priorities in the management of the pandemic. Management that should be oriented towards preserving the overall health and well-being of all populations, not ignoring those in conditions of vulnerability, and towards bringing its citizens together, not driving them apart.
